# Combining Transcriptomics and Proteomics Reveals Potential Post-transcriptional Control of Gene Expression After Light Exposure in *Metarhizium acridum*

**DOI:** 10.1534/g3.119.400430

**Published:** 2019-07-10

**Authors:** Guilherme T. P. Brancini, Márcia E. S. Ferreira, Drauzio E. N. Rangel, Gilberto Ú. L. Braga

**Affiliations:** * Faculdade de Ciências Farmacêuticas de Ribeirão Preto, Universidade de São Paulo, Ribeirão Preto, SP 14040-903, Brazil and; † Universidade Brasil, São Paulo, SP 08230-030, Brazil

**Keywords:** light, transcriptomics, proteomics, *Metarhizium*, stress

## Abstract

Light is an important stimulus for fungi as it regulates many diverse and important biological processes. *Metarhizium acridum* is an entomopathogenic fungus currently used for the biological control of insect pests. The success of this approach is heavily dependent on tolerance to environmental stresses. It was previously reported that light exposure increases tolerance to ultraviolet radiation in *M. acridum*. There is no information in the literature about how light globally influences gene expression in this fungus. We employed a combination of mRNA-Sequencing and high-throughput proteomics to study how light regulates gene expression both transcriptionally and post-transcriptionally. Mycelium was exposed to light for 5 min and changes at the mRNA and protein levels were followed in time-course experiments for two and four hours, respectively. After light exposure, changes in mRNA abundance were observed for as much as 1128 genes or 11.3% of the genome. However, only 57 proteins changed in abundance and at least 347 significant changes at the mRNA level were not translated to the protein level. We observed that light downregulated subunits of the eukaryotic translation initiation factor 3, the eIF5A-activating enzyme deoxyhypusine hydroxylase, and ribosomal proteins. We hypothesize that light is perceived as a stress by the cell that responds to it by reducing translational activity. Overall, our results indicate that light acts both as a signal and a stressor to *M. acridum* and highlight the importance of measuring protein levels in order to fully understand light responses in fungi.

*Metarhizium acridum* (Ascomycota: Sordariomycetes) is a soil-inhabiting entomopathogenic fungus currently used for the biological control of Orthoptera insects, mostly locusts and grasshoppers (Lacey *et al.* 2015). The success of biological control is heavily dependent on survival under harsh environmental conditions. Among these, heat and ultraviolet-B radiation (UV-B, 280-315 nm) are among the most stressful. The effects of UV-B radiation range from delayed conidia germination to complete inactivation (Braga *et al.* 2001; Braga *et al.* 2015). In this scenario, methods increasing *M. acridum* tolerance to UV-B radiation are highly sought after. Previous studies have shown that many physical and chemical factors can modulate stress tolerance in *Metarhizium* and other fungi (Rangel *et al.* 2011; Rangel *et al.* 2015; Dias *et al.* 2019). One of such factors is exposure to visible light.

Light is an important stimulus that regulates many biological processes in fungi. Depending upon the organism, light can regulate processes as diverse as development, secondary metabolite production, entrainment to circadian oscillators, and phototropism (Yu and Fischer 2019). Importantly, light responses are normally fast and transient with hierarchical signaling (Chen *et al.* 2009). Fungi respond to light by using photoreceptors capable of sensing mostly blue (phototropins), green (opsins), and red (phytochromes) light, although distinct fungi will differ in their ability to sense each of these wavelengths (Yu and Fischer 2019). In *Metarhizium robertsii*, growth under white light results in the production of conidia that germinate faster and are more virulent when compared to conidia produced in the dark (Oliveira *et al.* 2018). Also, using blue light during growth resulted in increased conidia yield (Oliveira *et al.* 2018). Regarding stress tolerance, we have previously reported that exposing *M. acridum* mycelium to white or blue light leads to increased tolerance to UV-B radiation (Brancini *et al.* 2016). We have also shown that light induces the expression of a photolyase gene and we and others have reported that photoreactivation is probably involved in UV-B radiation tolerance (Fang and St Leger 2012; Brancini *et al.* 2018). Nevertheless, we have no information about how light regulates gene expression genome-wide.

Genome-wide regulation after light exposure was evaluated in the ascomycete model *Neurospora crassa* and light was found to modulate the expression of as much as 24% of all predicted genes (Wu *et al.* 2014). However, the authors did not measure protein levels and therefore the number of changes at the mRNA level that are effectively translated to the protein level is still unknown. In this regard, a recent study focused on combining mRNA-Seq and high-throughput proteomics to study clock-controlled genes in *N. crassa* (Hurley *et al.* 2018). The authors observed that circadian output is highly influenced by post-transcriptional regulation, especially translational control, thus emphasizing the need to measure protein levels. Here we combined mRNA-Seq and Tandem Mass Tag (TMT)-based high-throughput proteomics to study how light regulates gene expression both transcriptionally and post-transcriptionally in *M. acridum*.

## Materials and Methods

### Strains and growth conditions

*Metarhizium acridum* ARSEF 324 was obtained from the USDA-ARS Collection of Entomopathogenic Fungal Cultures (Ithaca, NY, USA). The culture was maintained in Potato Dextrose Agar (Difco) supplemented with 0.5% yeast extract (Difco). Conidia were obtained by growing at 28° in complete darkness for 12 days.

### Light exposure

Conidia were scraped from plates and used to prepare a suspension at 2.5 × 10^7^ cells ml^-1^ in Tween 80 0.05% (Sigma). Four milliliters of this suspension were used to inoculate 100 ml of Potato Dextrose Broth (Difco) in 250 ml Erlenmeyer flasks. For each experiment, a total of six cultures were prepared. These cultures were grown in complete darkness at 28° under agitation (125 rpm) for 24 h to produce mycelium. Then, five of the six culture flasks were exposed to white light from fluorescent lamps (irradiance = 5.3 W m^−2^; photon flux = 24.7 µmol m^−2^ s) for 5 min. Flasks were moved back to dark for different lengths of time depending on experiment type. For transcriptomics, dark incubations after light exposure were for 0 (5L 0D), 10 (5L 10D), 25 (5L 25D), 55 (5L 55D), and 115 (5L 115D) min. For proteomics, these incubations were for 10 (5L 10D), 25 (5L 25D), 55 (5L 55D), 115 (5L 115D), and 235 (5L 235D) min. In both cases a control was always kept in the dark (DD). After the incubation was over, mycelium was vacuum filtered, washed with distilled water, and immediately frozen in liquid nitrogen. Frozen mycelia were stored at −70° until RNA or protein extraction. Three independent experiments were performed for mRNA-Seq and three independents experiments for high-throughput proteomics.

### Effects of light on the transcriptome

Frozen mycelia were ground with mortar and pestle under liquid nitrogen to obtain a fine powder. Approximately 50 mg of frozen powder were added to 450 µl RLT buffer from the RNeasy Plant Mini Kit (Qiagen). Purification was performed following manufacturer’s instructions and total RNA was eluted with nuclease-free water. Quality assessment was performed on an Agilent Bioanalyzer 2100 and all samples presented with RNA Integrity Number ≥ 7. Libraries were constructed with the TruSeq Stranded mRNA v4 (Illumina) following manufacturer’s instructions. Library quantification was performed via quantitative PCR and sequencing was run on HiSeq 2500 equipment. Three independent experiments were performed separately and sequenced together in the same lane. Because each experiment consisted of six samples, a total of 18 samples were sequenced yielding approximately 20 million reads per sample.

Sequencing data were aligned to *M. acridum* genome (Gao *et al.* 2011) with Hisat2 (Kim *et al.* 2015). The alignments were then analyzed with Cufflinks (Trapnell *et al.* 2010) using the -G option (no Reference Annotation Based Transcript assembly). Differential expression and statistical testing were performed with Cuffdiff 2 (Trapnell *et al.* 2013). Finally, Cuffdiff output was analyzed with cummeRbund (Trapnell *et al.* 2012). Differences between light treatments and DD were considered significant if they could satisfy *P* < 0.01 and a twofold cutoff. Gene clustering by expression pattern was performed with *clust* (Abu-Jamous and Kelly 2018), heat maps were built with TM4 MeV (Saeed *et al.* 2003), and principal component analysis was achieved with ClustVis (Metsalu and Vilo 2015). Gene ontology analyses were performed on the Blast2GO suite (Gotz *et al.* 2008).

Validation of mRNA-Seq data were performed for photolyase (MAC_05491) and UV-endonuclease (MAC_07337) coding genes with quantitative reverse transcription PCR (qRT-PCR). Total RNA extraction was performed exactly as described for mRNA-Seq and the downstream protocol for cDNA synthesis and gene quantification was as previously described (Brancini *et al.* 2018).

### Effects of light on the proteome

Frozen mycelia were ground with mortar and pestle under liquid nitrogen to obtain a fine powder. Approximately 50 mg of frozen powder were added to 500 µl of extraction buffer [7M urea, 2M thiourea, 4% CHAPS (Sigma)] and the mixture was vortexed for 2 min. Samples were then centrifuged at 10,000 × *g* and 4° for 5 min. The supernatant was collected and total protein was quantified with the 2-D Quant Kit (GE Healthcare). Protein purification was performed with a methanol/chloroform protocol as previously described (Wessel and Flugge 1984).

Proteins were reduced with dithiothreitol, alkylated with iodoacetamide, and finally digested with trypsin. Resulting peptides were labeled with TMT 10-plex (Thermo Scientific) with one tag for each condition according to manufacturer’s instructions. After isobaric tagging, the six conditions in each experiment were pooled and fractionated by reverse phase chromatography (C_18_, 1 × 100 mm, 3.5 μm, 130 Å, Waters). Elution was performed at 0.1 ml/min using a gradient of A (20 mM pH 10 ammonium formate) and B (acetonitrile) from 1 to 37.5% over 61 min. A total of 12 fractions were collected. These were dried in a vacuum centrifuge and solubilized in 0.1% formic acid.

Tandem mass spectrometry (MS/MS) analyses were performed as previously described (Becher *et al.* 2018). Briefly, peptides from each of the 12 fractions were analyzed on a nanoLC (UltiMate 300 RSLC, Thermo) equipped with a C_18_ pre-column (Precolumn C_18_ PepMap 100, 300 μm × 5 mm, 5 μm, 100 Å) and an analytical column (Acclaim C_18_ PepMap 100, 75 mm × 50 cm, 3 mm, 100 Å). The nanoLC equipment was coupled to a Q Exactive Plus Hybrid Quadrupole-Orbitrap mass spectrometer (Thermo). Elution was always performed with solvents A (0.1% formic acid in water) and B (0.1% formic acid in acetonitrile). Peptides were loaded into the column at 30 µl/min solvent A for 3 min. Peptides were eluted from the column with an elution gradient adjusted to 0.3 ml/min over 120 min. The concentration of B in the gradient was ramped to 4% over 4 min, to 8% over 2 min, to 26% over 96 min, and to 40% over 10 min. Eluted peptides were analyzed in positive mode and data-dependent method. Full scan spectra were obtained in the 375-12,000 m/z range. The top ten precursors in MS were selected for MS/MS.

Raw spectra were processed with IsobarQuant (Franken *et al.* 2015) and protein identification was performed with MASCOT (Matrix Science). Identification was based on the *M. acridum* genome (Gao *et al.* 2011). MASCOT search parameters were as follows: enzyme trypsin; up to three missed cleavages; peptide tolerance 10 ppm; MS/MS tolerance 0.02 Da; carbamidomethyl (Cys) and TMT10plex (Lys) as fixed modifications; TMT10plex on N-terminus, oxidation (Met), and N-acetylation as variable modifications. Batch effects were removed using limma (Ritchie *et al.* 2015) and results were normalized via the *vsn* strategy of variance normalization (Huber *et al.* 2002). Quantitative information was only analyzed when a given protein was found in two or three experiments. If the protein was identified in two experiments, missing data for the third experiment were imputed with the k-nearest neighbor algorithm. Changes at the protein level were considered significant if they could satisfy a twofold cutoff relative to DD at False Discovery Rate < 0.05. Combined mRNA/protein graphs were plotted with Origin 8.0 software (OriginLab Corporation).

### Data availability

Supplemental material available at Figshare: https://doi.org/10.25387/g3.8115998.

## Results

### Effects of light on the transcriptome

**Figure 1:**
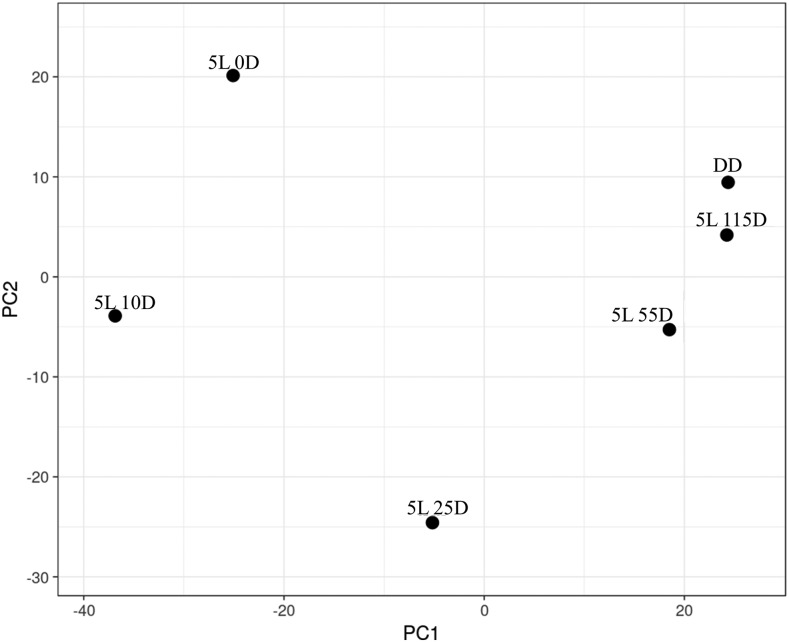
Principal component analysis for the 1128 light-regulated genes. Changes at mRNA level occurred mostly in the first 30 min after light exposure and not at later time points.

To evaluate light-regulated gene expression, we performed mRNA-Seq of RNA extracted from mycelia exposed to light for 5 min followed by incubation in the dark for different lengths of time (0, 10, 25, 55, and 115 min). A control was kept in complete darkness (DD). Our analysis encompassed 9514 genes corresponding to 95.4% of the genome (Table S1). A gene was considered light-regulated if significant mRNA change was observed in at least one time point relative to DD. Light regulated the expression of 4819 genes at *P* < 0.01. Because many genes were only weakly regulated, we applied a twofold cutoff and observed that 1128 transcripts changed in abundance under these criteria (Table S2). Of these, 719 (64%) were upregulated and 409 (36%) were downregulated. Principal component analysis revealed that the majority of changes occurred at the initial time points (especially 5L 0D, 5L 10D, and 5L 25D) and not at later time points (Figure 1).

**Figure 2:**
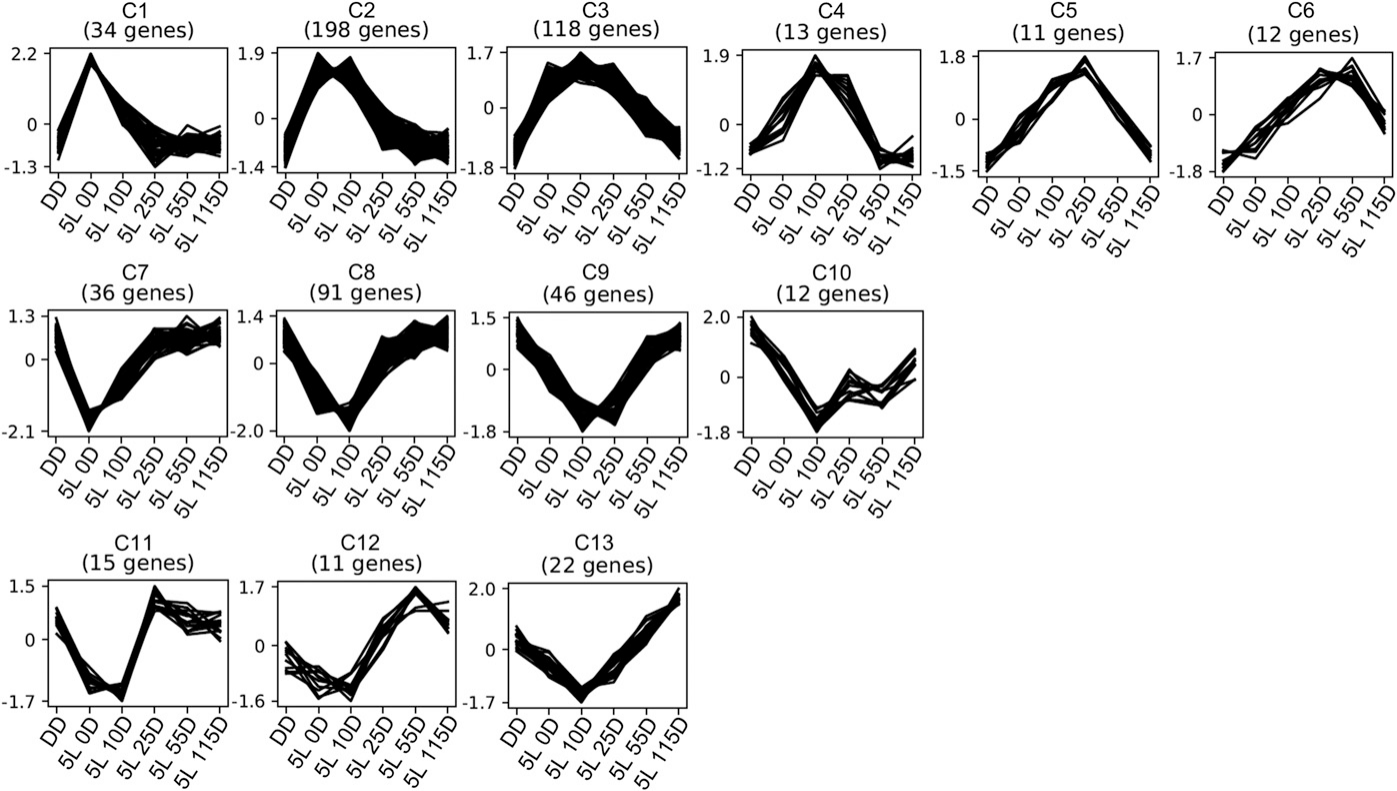
Clustering analysis for light-regulated genes resulted in 13 clusters depicting upregulated (C1 through C6), downregulated (C7 through C10), and oscillatory (C11 through C13) genes. Values in y-axis are Z-scores.

To understand the kinetics of gene regulation after light exposure, we clustered the 1128 light-regulated genes according to their expression profile by using *clust* (Abu-Jamous and Kelly 2018). *clust* deals with the clustering problem with a data extraction approach instead of the more traditional data partitioning. On the one hand, this generates tight clusters with little to no ambiguity in gene assignment. On the other hand, only about 50% of all genes are clustered (Abu-Jamous and Kelly 2018). For our data set, *clust* generated 13 clusters comprising 619 genes (54.9%) with an average cluster size of 47.6 genes (Figure 2 and Table S3).

The upregulated clusters (C1 through C6) showed that light can regulate gene expression at multiple time points, thus allowing us to classify genes as early- (5L 0D, 5L 10D, and 5L 25D; clusters C1 through C5) and late- (5L 55D and 5L 115D; cluster C6) regulated according to their peak expression (Figure 2). Also, this revealed a potential hierarchical model in which light initially drives the expression of genes coding for transcription factors that will then act on downstream genes. Approximately the same phenomenon was observed for downregulated gene clusters (C7 through C10), although late downregulated genes were not observed (Figure 2). Finally, some gene clusters presented an oscillatory pattern characterized by initial downregulation followed by late upregulation (C11 through C13) (Figure 2).

**Figure 3:**
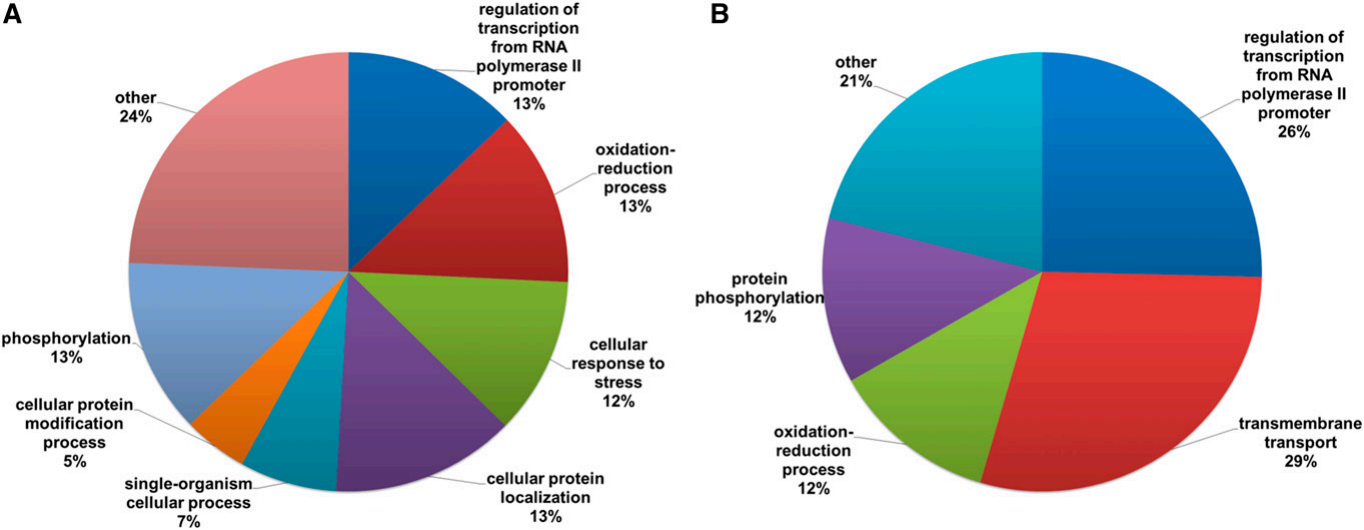
Gene Ontology analysis for genes on clusters (A) C2 and (B) C8 which are the largest up and downregulated gene clusters, respectively.

To gain better insight into which biological processes were regulated by light, we performed Gene Ontology analyses on clusters C2 and C8 which are the largest up and downregulated gene clusters, respectively. Overall, light upregulated genes involved in cellular response to stress and cellular protein localization (Figure 3A) and downregulated genes involved in transmembrane transport (Figure 3B). Some biological processes, such as ‘oxidation-reduction process’ and ‘regulation of transcription from RNA polymerase II promoter’, were shared by both clusters. Because response to stress and transcriptional regulation were enriched in cluster C2, we looked for genes belonging to known oxidative stress response pathways. We observed that a stress-activated MAPK gene (MAC_08084) homolog to *N. crassa os-2* and *Aspergillus nidulans hogA* was upregulated together with the bZip transcription factor *asl-1* homolog (MAC_03844).

**Figure 4:**
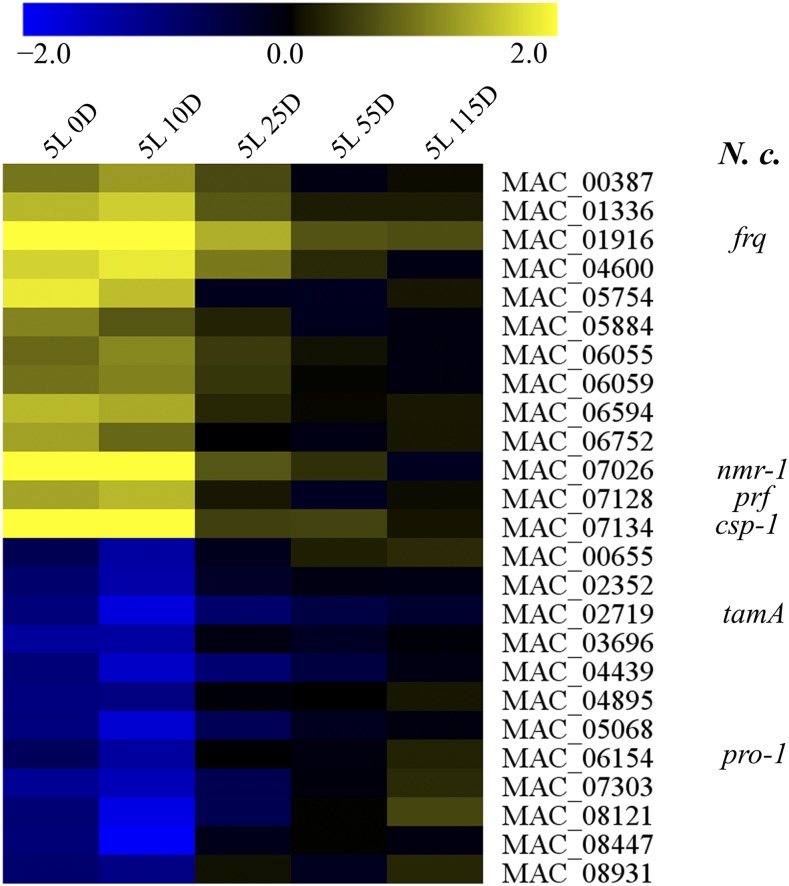
Heat map depicting the early regulation of genes coding for transcriptional regulators on clusters C2 and C8. Transcriptional regulator activity was according to Gene Ontology. Values in scale bar are log_2_ fold-change relative to DD. *N . c*. = *Neurospora crassa* known homolog genes.

Because transcriptional regulators were abundant in clusters C2 and C8, we performed a separate analysis for such light-regulated genes (Figure 4). Among these, we found homolog genes for the core circadian oscillator *frq* (MAC_01916) and the circadian transcriptional repressor *csp-1* (MAC_07134) both of which are also regulated by light in *N. crassa* (Froehlich *et al.* 2002; Sancar *et al.* 2011). Future experiments should elucidate whether *M. acridum* possesses a circadian clock.

**Figure 5:**
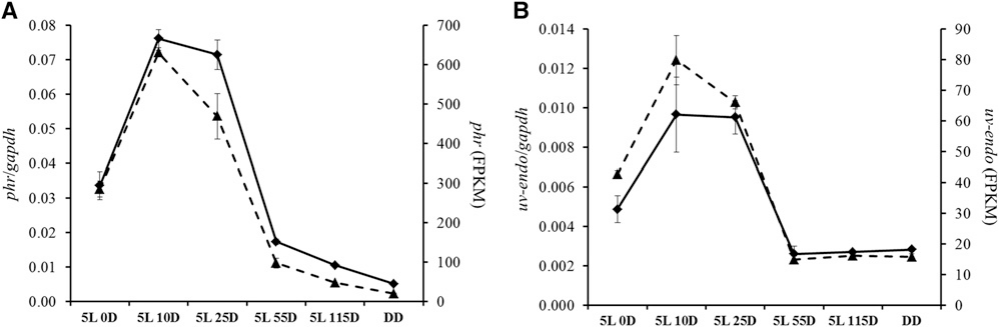
Validation of mRNA-Seq data for the (A) *phr* (MAC_05491) and (B) *uv-endo* (MAC_07337) genes with quantitative reverse transcription PCR (qRT-PCR). Solid lines refer to qRT-PCR (primary *y*-axis) and dashed lines refer to mRNA-Seq data (secondary *y*-axis). Error bars are standard deviation from three independent experiments.

For validation purposes, we have evaluated the expression of genes encoding for a photolyase (MAC_05491) and a UV-endonuclease (MAC_07337) by qRT-PCR. Similar patterns of light regulation in mRNA-Seq and qRT-PCR experiments were observed for both genes (Figure 5).

### Effects of light on the proteome

**Figure 6:**
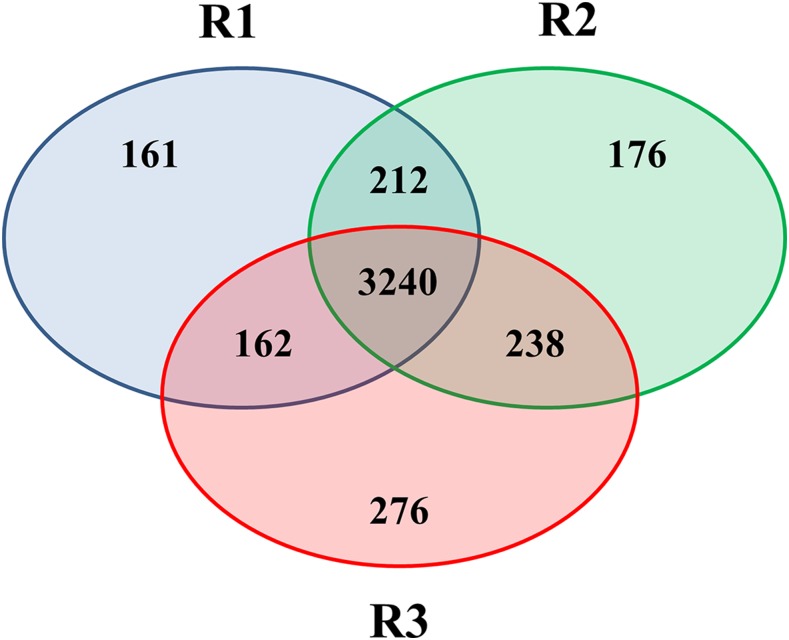
Venn diagram showing the number of identified proteins in experiments R1, R2, and R3. Quantitative data were only used if a given protein was identified in at least two experiments, resulting in quantitative information for 3852 proteins.

For high-throughput proteomics experiments, we analyzed a longer time point (5L 235D) in order to better account for the expected delay between mRNA and protein peak. We also removed the very short 5L 0D time point from proteomics analyses. Our proteomics data showed good agreement between the three experiments and quantitative information was used only if a protein was present in at least two experiments (Figure 6).

**Figure 7:**
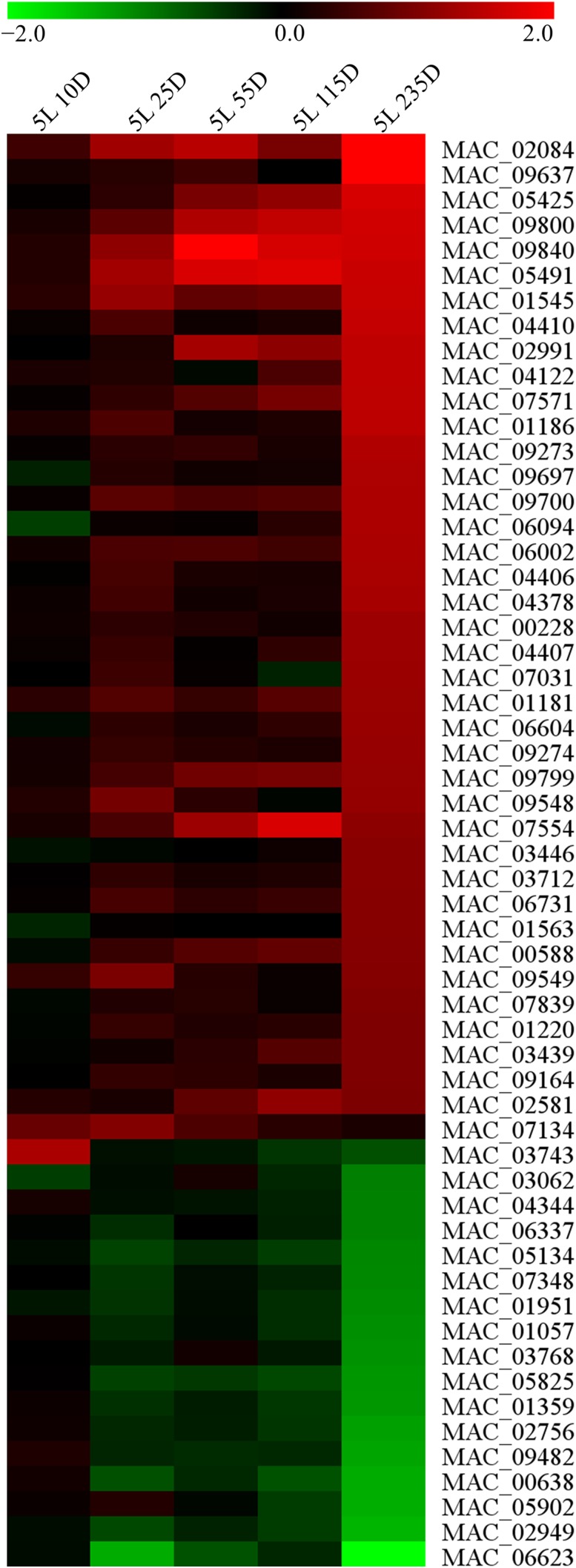
Heat map depicting changes at the protein level after light exposure. Almost 90% of all proteins were regulated at 5L 235D. Values in scale bar are log_2_ fold-change relative to DD.

Our analysis encompassed 3852 proteins representing 38.6% of all predicted gene products. Of these, only 57 were regulated by light at least twofold, with 41 upregulated and 16 downregulated proteins. Changes in abundance at the protein level peaked at 5L 235D for 89.5% of regulated proteins, with only six proteins changing at earlier time points (Figure 7).

We then analyzed the top 10 most up and downregulated proteins after light exposure (Table 1 and Table 2). The strongest upregulated protein, acid sphingomyelinase (MAC_02084), is involved in sphingolipid metabolism. SignalP-5.0 (Almagro Armenteros *et al.* 2019) sequence analysis revealed the presence of an N-terminal secretory signal peptide that could indicate the protein has a role in insect pathogenicity. Five out of the ten most upregulated proteins are currently uncharacterized and two of these (MAC_09637, MAC_02991) have no homologs in *N. crassa*. One of the uncharacterized proteins (MAC_09800) is annotated as a flavin-binding monooxygenase in *M. guizhouense*, *M. brunneum*, and *M. majus*. We also observed the accumulation of two other flavin-binding monooxygenases (MAC_09799, MAC_09164) after light exposure (Table S4). Furthermore, MAC_09799 and MAC_09800 are neighboring genes and presented the same protein accumulation profile (Table S4). We also observed the upregulation of heat shock protein 30 (MAC_07554) and photolyase (MAC_05491), both probably involved in light-induced stress tolerance.

**Table 1:** Ten most upregulated proteins after light exposure

	Name	Gene ID	Protein Entry	log_2_ fold-change*^a^* (time point)
**1**	Acid sphingomyelinase, putative	MAC_02084	E9DWT6	2.40 (5L 235D)
**2**	Uncharacterized protein	MAC_09637	E9EID9	2.09 (5L 235D)
**3**	Membrane protein, putative	MAC_09840	E9EIZ2	2.00 (5L 55D)
**4**	Photolyase	MAC_05491	E9E6J3	1.76 (5L 115D)
**5**	Heat shock protein 30	MAC_07554	E9ECF6	1.70 (5L 115D)
**6**	Uncharacterized protein	MAC_05425	E9E6C7	1.69 (5L 235D)
**7**	Uncharacterized protein	MAC_09800	E9EIV2	1.64 (5L 235D)
**8**	Uncharacterized protein	MAC_01545	E9DV97	1.57 (5L 235D)
**9**	Lysine amidinotransferase	MAC_04410	E9E3G2	1.56 (5L 235D)
**10**	Uncharacterized protein	MAC_02991	E9DZE3	1.51 (5L 235D)

a peak log_2_ fold-change relative to DD.

**Table 2:** Ten most downregulated proteins after light exposure

	Name	Gene ID	Protein Entry	log_2_ fold-change*^a^* (time point)
**1**	Cytochrome P450 phenylacetate 2-hydroxylase, putative	MAC_06623	E9E9S5	−2.17 (5L 235D)
**2**	Amino acid transporter, putative	MAC_02949	E9DZA1	−1.42 (5L 235D)
**3**	Carboxyphosphonoenolpyruvate phosphonomutase, putative	MAC_05902	E9E7Q4	−1.37 (5L 235D)
**4**	54S ribosomal protein L12	MAC_00638	E9DSP0	−1.35 (5L 235D)
**5**	Eukaryotic translation initiation factor 3 subunit E	MAC_09482	E9EHY4	−1.30 (5L 235D)
**6**	GNAT family N-acetyltransferase, putative	MAC_02756	E9DYQ9	−1.26 (5L 235D)
**7**	Deoxyhypusine hydroxylase	MAC_01359	E9DUR1	−1.18 (5L 235D)
**8**	Vitamin B6 transporter, putative	MAC_05825	E9E7H7	−1.18 (5L 235D)
**9**	Rhomboid family protein	MAC_03768	E9E1M0	−1.15 (5L 235D)
**10**	Eukaryotic translation initiation factor 3 subunit M	MAC_01057	E9DTV9	−1.12 (5L 235D)

a peak log_2_ fold-change relative to DD.

**Figure 8:**
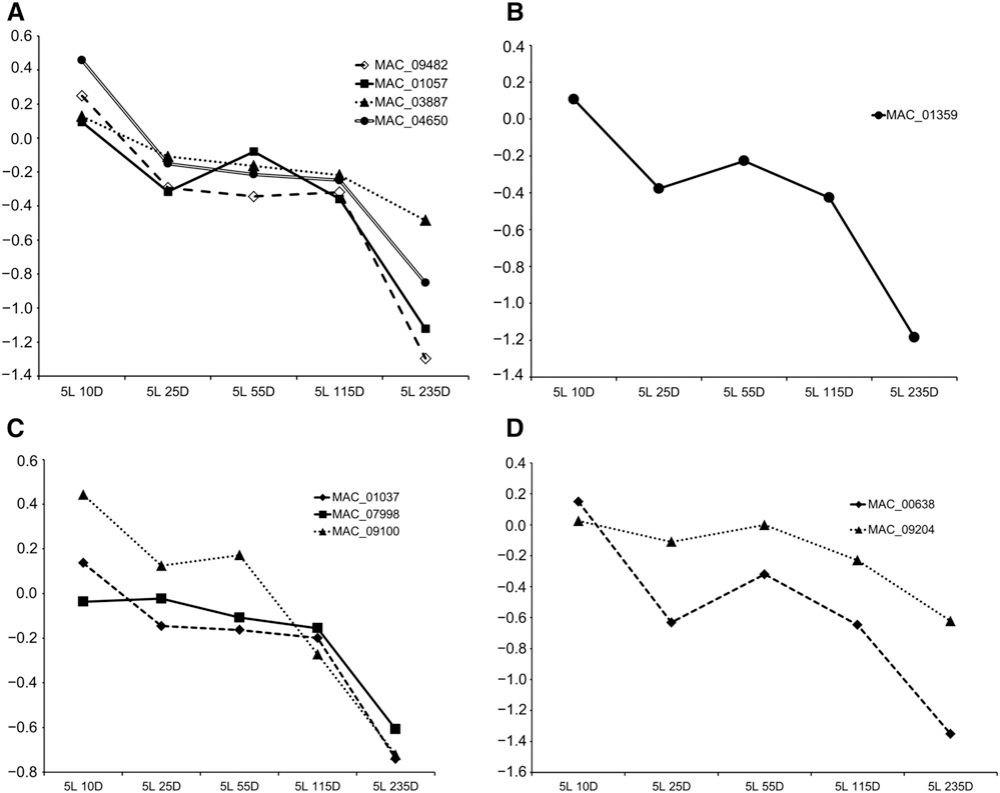
Light downregulated proteins involved in translation, including (A) eIF3 subunits M (MAC_01057), E (MAC_09482), K (MAC_04650), and F (MAC_03887); (B) the eIF5A-activating enzyme deoxyhypusine hydroxylase (MAC_01359); (**C**) cytosolic ribosomal proteins P0 (MAC_01037), S14 (MAC_07998), and S29 (MAC_09100); and (D) mitochondrial ribosomal proteins L12 (MAC_00638) and MRP2 (MAC_09204). Values in y-axis are log_2_ fold-change relative to DD.

Among downregulated proteins, subunits E and M of eukaryotic translation initiation factor 3 (eIF3) were at least twofold regulated after light exposure (Table 2). The downregulation of two eIF3 subunits prompted us to lower the twofold cutoff in the search for other regulated eIF3 subunits. We found eIF3 subunit K to be 1.8-fold and eIF3 subunit F to be 1.4-fold downregulated (Figure 8A). This observation was specific to eIF3 as subunits for other translation initiation factors were unchanged (Table S5). However, the enzyme deoxyhypusine hydroxylase (MAC_01359) was downregulated at the protein level (Table 2 and Figure 8B). This protein is one of two enzymes required for the post-translational modification that activates eukaryotic initiation factor 5A (eIF5A) which has a role in translation elongation (Saini *et al.* 2009).

The decreased translation initiation/elongation caused by light exposure prompted us to look for regulated ribosomal proteins. We observed downregulation of 40S ribosomal proteins S14 and S29 (MAC_07998, MAC_09100) and 60S ribosomal protein P0 (MAC_01037), although these only satisfied a 1.5-fold cutoff (Figure 8C). Furthermore, mitochondrial ribosomal proteins 54S L12 (MAC_00638) and 40S MRP2 (MAC_09204) were 2.5- and 1.5-fold downregulated, respectively (Table 2 and Figure 8D).

### Combining proteomics and mRNA-Seq data to find post-transcriptional regulatory mechanisms

After light exposure, 1128 mRNAs (out of 9514 evaluated) changed in abundance while only 57 proteins (out of 3852 evaluated) did so. Combining both data sets resulted in 34 light-regulated mRNA/protein pairs. We used these pairs to elucidate the average time required to go from peak mRNA to peak protein change. This was done by calculating R^2^ for log_2_-log_2_ correlation plots. Overall, mRNA change at any time point best correlated with protein change 1-2 h later (Table 3 and Table S6).

**Table 3:** Person correlation coefficient for changes at the mRNA and protein levels. Correlation was calculated based on the 34 light-regulated mRNA/protein pairs.

		Proteomics				
		5L 10D	5L 25D	5L 55D	5L 115D	5L 235D
**mRNA-Seq**	5L 0D	0.29	0.26	0.74	0.73	0.10
	5L 10D	0.15	0.11	0.71	0.70	0.02
	5L 25D	0.11	0.19	0.79	0.76	0.16
	5L 55D	0.18	0.21	0.78	0.81	0.36
	5L 115D	0.04	0.28	0.55	0.59	0.65

**Figure 9:**
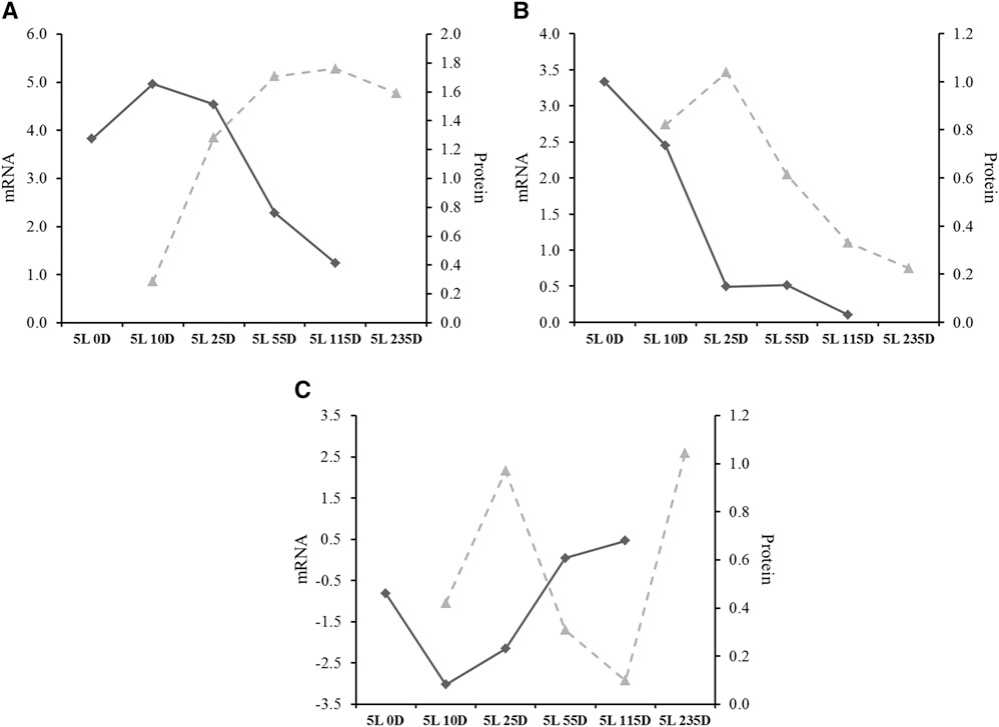
Combined mRNA/protein plots showing good correlation for (A) photolyase (MAC_05491) and (B) C2H2 transcription factor (= *N. crassa* CSP-1, MAC_07134) with an inverse and poor correlation for (C) polyketide synthase (MAC_09549). Solid lines refer to mRNA and dashed lines refer to protein. Values in *y*-axes are log_2_ fold-change relative to DD.

The majority of pairs followed this 1-2 h delay as observed for the photolyase (Figure 9A). A very early regulated gene coding for a C2H2 transcription factor (= *N. crassa* CSP-1) presented an accompanying early protein accumulation and was one of the fastest regulated protein in the data set, perhaps a requirement to fulfill its biological role (Figure 9B). In at least two instances there was protein accumulation after gene downregulation, such as observed for a polyketide synthase (Figure 9C).

A consequence of having only 34 mRNA/protein pairs is that 23 proteins changed abundance in the absence of mRNA regulation (Table 4). In principle, this would leave us with 1094 mRNAs for which there was no protein change. However, we need to take into account that mRNA-Seq and proteomic data sets are different sizes (9514 *vs.* 3852, Table 4). Therefore, the number of mRNAs changing after light exposure without an accompanying protein change is actually 347, while the remaining 747 present no protein quantitative data (Table 4). This means that a large number of changes at the mRNA level are not translated into changes at the protein level. More importantly, it was not possible to predict, based on mRNA fold change or expression pattern, whether regulation at the transcript level would lead to changes at the protein level. Some mRNAs were upregulated by as much as 18-fold and downregulated by as much as 5.7-fold without any accompanying changes in protein expression levels (Table S6).

**Table 4:** Combination of mRNA-Seq and proteomics data sets based on the number of regulated mRNAs/proteins

	mRNA-Seq	Proteomics
**Genome**	9974	
**Evaluated mRNAs/proteins**	9514	3852
**Light-regulated**	1128*^a^*	57*^b^*
**Upregulated**	719 (64%)	41 (72%)
**Downregulated**	409 (36%)	16 (28%)
**mRNA/protein pairs**	34	
**protein change without mRNA change**	23	
**mRNA change without protein change**	347	

a *P* < 0.01 and at least twofold regulation.

b False Discovery Rate < 0.05 and at least twofold regulation.

## Discussion

The success of biological control with *M. acridum* depends on the fungus surviving the stresses imposed by the environment. Among these, heat and UV-B radiation can be cited as the most relevant. We previously observed that a 5-min exposure to light will increase tolerance to UV-B radiation in a time-dependent manner (Brancini *et al.* 2016). Therefore, we combined transcriptomics (mRNA-Seq) and high-throughput proteomics to understand how light regulates gene expression both transcriptionally and post-transcriptionally. Our experiments were performed by exposing mycelium to a 5-min pulse of light and then incubating it in the dark for different lengths of time. Conversely, most studies evaluating light responses in fungi expose mycelium to light for different lengths of time with no incubation in the dark afterward (Chen *et al.* 2009; Ruger-Herreros *et al.* 2011; Fuller *et al.* 2013; Schumacher *et al.* 2014; Wu *et al.* 2014). Because light is also regarded as a stress to fungi, different exposures to light would inevitably lead to varying amounts of stress based on the length of light exposure. We have therefore tried to mitigate this effect by using the same exposure for all time points in our analysis. Furthermore, the 5-min exposure was chosen based on prior work (Brancini *et al.* 2016) in which we observed that this exposure was sufficient to result in increased tolerance to UV-B radiation.

Light transcriptionally regulated 1128 genes or 11.3% of the genome after a 5-min exposure. Most genes were regulated in the first 30 min after light exposure with only few genes being late regulated (Figure 1 and 2). According to the hierarchical model of gene regulation by light uncovered in *N. crassa* (Chen *et al.* 2009), the White Collar Complex (WCC) initially drives the expression of its target genes and some of these are transcription factors that will then act downstream of the WCC to regulate other genes. Accordingly, we observed early regulation of many genes coding for transcriptional regulators (Figure 4). We hypothesize that the reduced number of late regulated genes is a consequence of the short initial exposure to light. Longer exposures would keep gene expression levels higher instead of creating a quick rise and fall in mRNA abundance as observed in our data (Figure 2) and this could be essential for the induction of late regulated genes. A previous work employed mRNA-Seq to study light regulation in *N. crassa* by exposing mycelium to light for 0, 15, 60, 120, and 240 min (Wu *et al.* 2014). Light was kept on throughout the experiment (no dark incubation afterward) and the authors observed consistent gene regulation at all time points. This supports the hypothesis that late gene regulation could also be dependent on longer exposures to light in *M. acridum*.

The strongest upregulated protein in our data set was an acid sphingomyelinase (Table 1), responsible for the breakdown of sphingomyelin to ceramide and phosphorylcholine. A gene coding for a sphingomyelinase was upregulated in *Ophiocordyceps unilateralis s.l*. during ant infection (de Bekker *et al.* 2015). The authors speculate that a secreted sphingomyelinase could be involved in insect behavior manipulation by regulating sphingolipid metabolism. Insect behavior control by *M. acridum* has never been observed, but the strong accumulation of a sphingomyelinase containing an export signal peptide could indicate it has a role in insect killing as observed for *Bacillus cereus* (Doll *et al.* 2013). The accumulation of the enzyme in response to light is in accordance with the host lifestyle, as locusts are known to engage in behavioral fever by basking in the sun, a phenomenon beginning on day 1 post-infection (Clancy *et al.* 2018).

Proteins also upregulated after light exposure were a photolyase (Table 1) and a Cry-DASH (Table S4). We have previously reported that light increased photoreactivating ability in *M. acridum* and hypothesized that photoreactivation mediated UV-B radiation tolerance (Brancini *et al.* 2018). While both proteins were upregulated, the photolyase accumulated much faster than the cryptochrome. The former surpassed the twofold cutoff at 5L 25D whereas the latter only did so at 5L 235D. Because tolerance to UV-B radiation increases quickly after light exposure, the photolyase is possibly the better candidate enzyme for mediating photoreactivation and UV-B tolerance with Cry-DASH fulfilling other regulatory roles as observed in *A. nidulans* and *Fusarium fujikuroi* (Bayram *et al.* 2008; Castrillo *et al.* 2013). It is important to note that the photolyase accumulating after light exposure is the same for which we observed photoinduction at the mRNA level in our previous publication (Brancini *et al.* 2018).

Interestingly, many proteins negatively regulated by light were found to be involved in translation (Table 2 and Table S4). Downregulation of eIF3 subunits (Figure 8A) and of the eIF5A-activating enzyme deoxyhypusine hydroxylase (Figure 8B) suggests that light exposure reduces translational activity by acting on both translation initiation and elongation. Furthermore, some cytosolic and mitochondrial ribosomal proteins were also downregulated (Figure 8C and 8D). Surprisingly, this potential reduction in translational activity peaked at 5L 235D, when almost all changes in protein abundance were observed (Figure 7). Translation reduction and reprogramming are known cellular responses to stress (Yamasaki and Anderson 2008; Spriggs *et al.* 2010; Crawford and Pavitt 2019). Also, decreased expression of genes coding for ribosomal proteins was observed after *N. crassa* mycelium was exposed to light (Wu *et al.* 2014). Here we show that this phenomenon is also observable at the protein level which is in agreement with the idea that light serves as both a signal and a stress to the cell (Wu *et al.* 2014). Recently, Hurley and coworkers have shown that translation in *N. crassa* is under the influence of the circadian clock and occurs preferentially after dusk and not during the day (Hurley *et al.* 2018), which is line with prior work reporting that translational activity is decreased at late subjective morning (Caster *et al.* 2016). It seems reasonable to say that light reduces translational activity in *N. crassa* by resetting the clock to subjective morning. It should be noted that the aforementioned downregulated proteins did not present downregulation of their corresponding mRNAs in our data set. This could be due to these proteins being post-transcriptionally regulated or it could be a consequence of late gene regulation that is beyond our last time point (5L 115D).

Combining our transcriptomic and proteomic data sets revealed an interesting phenomenon: while 1128 mRNAs changed in abundance in response to light, only 57 proteins did so (Table 4). These values correspond to 11.8% of all 9514 evaluated transcripts and 1.48% of all 3852 evaluated proteins. No more than 34 mRNA-protein pairs could be formed that were regulated in both data sets (Table S6). These pairs were used to calculate the 1-2 h delay required to go from mRNA peak regulation to protein peak regulation (Figure 9 and Table 3).

As mentioned above, we observed that the majority of changes at the mRNA level were not translated to changes at the protein level. Lack of protein change in the event of gene downregulation could be explained by protein stability: stable proteins will last many hours inside the cell and our experiment only encompassed the first four hours following light exposure. However, if proteins are stable, then accumulation would be expected from gene upregulation. The negative effect light apparently had on translation initiation and elongation could perhaps help explain such a phenomenon.

Under conditions of decreased translational activity, there should be a mechanism allowing specific mRNAs to bypass this overall decrease. Light downregulated eIF3 subunits E (eIF3e), M (eIF3m), K (eIF3k), and F (eIF3f) but it did not regulate any other subunit. eIF3 is thought to mediate 43S pre-initiation complex assembly and attachment to mRNA, scanning, and start codon selection (Hinnebusch 2017). In *Schizosaccharomyces pombe*, there are two distinct eIF3 complexes formed with either eIF3m or eIF3e (Zhou *et al.* 2005). On the one hand, the complex formed with eIF3m binds to the bulk of cellular mRNA and is responsible for overall translation. This makes *eIF3m* an essential gene. On the other hand, the complex formed with eIF3e is more restricted and regulates the translation of specific mRNAs (Zhou *et al.* 2005). In *N. crassa*, mutants for all the known eIF3 subunits were analyzed. In accordance with *S. pombe*, *eIF3m* was found to be an essential gene whereas *eIF3e* and *eIF3k* mutants were viable (Smith *et al.* 2013). Therefore, different eIF3 subunits are probably involved in the translation of distinct mRNA molecules and their regulation constitutes an additional layer of post-transcriptional control (Genuth and Barna 2018). We speculate that light can affect the translation of specific mRNAs by regulating eIF3 subunits and therefore translation initiation.

In line with this hypothesis, light also downregulated some ribosomal proteins (Figure 8C and 8D) while the majority remained unchanged (Table S5). It was previously shown in mouse embryonic stem cells that active ribosomes are heterogeneous with respect to ribosomal proteins (Shi *et al.* 2017). These heterogeneous ribosomes translate different pools of mRNAs involved in different biological processes such as metabolism, proliferation, and cell survival. For instance, RPL10A was found to be required for the translation of specific mRNAs. This regulation was mediated, at least in part, by 5′ UTR internal ribosome entry site (IRES) elements (Shi *et al.* 2017). It seems a natural consequence that regulating the abundance of ribosomal proteins could lead to differences in mRNA translation for specific genes sets. This “ribosome code” has been speculated and discussed for the past 60 years, but it is only recently gaining more attention (Emmott *et al.* 2019).

Taken together, our results indicate that light acts as both a signal and a stress in *M. acridum*. When acting as a signal, light regulates the transcription of as much as 11.3% of the genome. Because it is also perceived as a stress, light ultimately causes a decrease in translational activity by downregulating some eIF3 subunits, the eIF5A-activating enzyme deoxyhypusine hydroxylase, and ribosomal proteins. We hypothesize the downregulation of these proteins buffers the changes at the mRNA level and ultimately results in the small number of regulated proteins observed. Therefore, our results show that changes at the mRNA level are not necessarily translated to changes at the protein level and highlight the importance of analyzing the proteome in order to fully understand light responses in fungi.

## References

[bib1] Abu-Jamous B., and Kelly S., 2018 Clust: automatic extraction of optimal co-expressed gene clusters from gene expression data. Genome Biol. 19: 172 10.1186/s13059-018-1536-8 30359297 PMC6203272

[bib2] Almagro Armenteros J. J., Tsirigos K. D., Sonderby C. K., Petersen T. N., Winther O., 2019 SignalP 5.0 improves signal peptide predictions using deep neural networks. Nat. Biotechnol. 37: 420–423. 10.1038/s41587-019-0036-z 30778233

[bib3] Bayram Ö., Biesemann C., Krappmann S., Galland P., and Braus G. H., 2008 More Than a Repair Enzyme: *Aspergillus nidulans* Photolyase-like CryA Is a Regulator of Sexual Development. Mol. Biol. Cell 19: 3254–3262. 10.1091/mbc.e08-01-0061 18495868 PMC2488289

[bib4] Becher I., Andrés-Pons A., Romanov N., Stein F., Schramm M., 2018 Pervasive Protein Thermal Stability Variation during the Cell Cycle. Cell 173:1495–1507 e1418. 10.1016/j.cell.2018.03.053 PMC5998384 29706546

[bib5] Braga G. U., Flint S. D., Miller C. D., Anderson A. J., and Roberts D. W., 2001 Both solar UVA and UVB radiation impair conidial culturability and delay germination in the entomopathogenic fungus *Metarhizium anisopliae*. Photochem. Photobiol. 74: 734–739. 10.1562/0031-8655(2001)074<0734:BSUAUR>2.0.CO;2 11723803

[bib6] Braga G. U., Rangel D. E., Fernandes E. K., Flint S. D., and Roberts D. W., 2015 Molecular and physiological effects of environmental UV radiation on fungal conidia. Curr. Genet. 61: 405–425. 10.1007/s00294-015-0483-0 25824285

[bib7] Brancini G. T., Rangel D. E., and Braga G. U., 2016 Exposure of *Metarhizium acridum* mycelium to light induces tolerance to UV-B radiation. FEMS Microbiol. Lett. 363 10.1093/femsle/fnw036 26884481

[bib8] Brancini G. T. P., Bachmann L., Ferreira M., Rangel D. E. N., and Braga G. U. L., 2018 Exposing *Metarhizium acridum* mycelium to visible light up-regulates a photolyase gene and increases photoreactivating ability. J. Invertebr. Pathol. 152: 35–37. 10.1016/j.jip.2018.01.007 29408156

[bib9] Caster S. Z., Castillo K., Sachs M. S., and Bell-Pedersen D., 2016 Circadian clock regulation of mRNA translation through eukaryotic elongation factor eEF-2. Proc. Natl. Acad. Sci. USA 113: 9605–9610. 10.1073/pnas.1525268113 27506798 PMC5003280

[bib10] Castrillo M., García-Martínez J., and Avalos J., 2013 Light-Dependent Functions of the *Fusarium fujikuroi* CryD DASH Cryptochrome in Development and Secondary Metabolism. Appl. Environ. Microbiol. 79: 2777–2788. 10.1128/AEM.03110-12 23417004 PMC3623198

[bib11] Chen C. H., Ringelberg C. S., Gross R. H., Dunlap J. C., and Loros J. J., 2009 Genome-wide analysis of light-inducible responses reveals hierarchical light signalling in *Neurospora*. EMBO J. 28: 1029–1042. 10.1038/emboj.2009.54 19262566 PMC2683703

[bib12] Clancy L. M., Jones R., Cooper A. L., Griffith G. W., and Santer R. D., 2018 Dose-dependent behavioural fever responses in desert locusts challenged with the entomopathogenic fungus *Metarhizium acridum*. Sci. Rep. 8: 14222 10.1038/s41598-018-32524-w 30242193 PMC6155106

[bib13] Crawford R. A. and Pavitt G. D., 2019 Translational regulation in response to stress in *Saccharomyces cerevisiae*. Yeast 36: 5–21. 10.1002/yea.3349 30019452 PMC6492140

[bib14] de Bekker C., Ohm R. A., Loreto R. G., Sebastian A., Albert I., 2015 Gene expression during zombie ant biting behavior reflects the complexity underlying fungal parasitic behavioral manipulation. BMC Genomics 16: 620 10.1186/s12864-015-1812-x 26285697 PMC4545319

[bib15] Dias L. P., Pedrini N., Braga G. U. L., Ferreira P. C., Pupin B., 2019 Outcome of blue, green, red, and white light on *Metarhizium robertsii* during mycelial growth on conidial stress tolerance and gene expression. Fungal Biol. 10.1016/j.funbio.2019.04.007 32389288

[bib16] Doll V. M., Ehling-Schulz M., and Vogelmann R., 2013 Concerted Action of Sphingomyelinase and Non-Hemolytic Enterotoxin in Pathogenic *Bacillus cereus*. PLoS One 8: e61404 10.1371/journal.pone.0061404 23613846 PMC3628865

[bib17] Emmott E., Jovanovic M., and Slavov N., 2019 Ribosome Stoichiometry: From Form to Function. Trends Biochem. Sci. 44: 95–109. 10.1016/j.tibs.2018.10.009 30473427 PMC6340777

[bib18] Fang W., and St Leger R. J., 2012 Enhanced UV resistance and improved killing of malaria mosquitoes by photolyase transgenic entomopathogenic fungi. PLoS One 7: e43069 10.1371/journal.pone.0043069 22912789 PMC3422317

[bib19] Franken H., Mathieson T., Childs D., Sweetman G. M., Werner T., 2015 Thermal proteome profiling for unbiased identification of direct and indirect drug targets using multiplexed quantitative mass spectrometry. Nat. Protoc. 10: 1567–1593. 10.1038/nprot.2015.101 26379230

[bib20] Froehlich A. C., Liu Y., Loros J. J., and Dunlap J. C., 2002 White Collar-1, a circadian blue light photoreceptor, binding to the frequency promoter. Science 297: 815–819. 10.1126/science.1073681 12098706

[bib21] Fuller K. K., Ringelberg C. S., Loros J. J., and Dunlap J. C., 2013 The fungal pathogen *Aspergillus fumigatus* regulates growth, metabolism, and stress resistance in response to light. MBio 4 10.1128/mBio.00142-13 PMC3604765 23532976

[bib22] Gao Q., Jin K., Ying S. H., Zhang Y., Xiao G., 2011 Genome Sequencing and Comparative Transcriptomics of the Model Entomopathogenic Fungi *Metarhizium anisopliae* and *M. acridum*. PLoS Genet. 7: e1001264 10.1371/journal.pgen.1001264 21253567 PMC3017113

[bib23] Genuth N. R., and Barna M., 2018 Heterogeneity and specialized functions of translation machinery: from genes to organisms. Nat. Rev. Genet. 19: 431–452. 10.1038/s41576-018-0008-z 29725087 PMC6813789

[bib24] Gotz S., Garcia-Gomez J. M., Terol J., Williams T. D., Nagaraj S. H., 2008 High-throughput functional annotation and data mining with the Blast2GO suite. Nucleic Acids Res. 36: 3420–3435. 10.1093/nar/gkn176 18445632 PMC2425479

[bib25] Hinnebusch A. G., 2017 Structural Insights into the Mechanism of Scanning and Start Codon Recognition in Eukaryotic Translation Initiation. Trends Biochem. Sci. 42: 589–611. 10.1016/j.tibs.2017.03.004 28442192

[bib26] Huber W., von Heydebreck A., Sultmann H., Poustka A., and Vingron M., 2002 Variance stabilization applied to microarray data calibration and to the quantification of differential expression. Bioinformatics 18: S96–S104. 10.1093/bioinformatics/18.suppl_1.S96 12169536

[bib27] Hurley J. M., Jankowski M. S., De Los Santos H., Crowell A. M., Fordyce S. B., 2018 Circadian Proteomic Analysis Uncovers Mechanisms of Post-Transcriptional Regulation in Metabolic Pathways. Cell Syst. 7: 613–626.e5. 10.1016/j.cels.2018.10.014 30553726 PMC6433121

[bib28] Kim D., Langmead B., and Salzberg S. L., 2015 HISAT: a fast spliced aligner with low memory requirements. Nat. Methods 12: 357–360. 10.1038/nmeth.3317 25751142 PMC4655817

[bib29] Lacey L. A., Grzywacz D., Shapiro-Ilan D. I., Frutos R., Brownbridge M., 2015 Insect pathogens as biological control agents: Back to the future. J. Invertebr. Pathol. 132: 1–41. 10.1016/j.jip.2015.07.009 26225455

[bib30] Metsalu T., and Vilo J., 2015 ClustVis: a web tool for visualizing clustering of multivariate data using Principal Component Analysis and heatmap. *Nucleic Acids Res* 43 (Web Server issue):W566–570. https://doi.org/10.1093/nar/gkv468 10.1093/nar/gkv468 PMC4489295 25969447

[bib31] Oliveira A. S., Braga G. U. L., and Rangel D. E. N., 2018 *Metarhizium robertsii* illuminated during mycelial growth produces conidia with increased germination speed and virulence. Fungal Biol. 122: 555–562. 10.1016/j.funbio.2017.12.009 29801800

[bib32] Rangel D. E., Braga G. U., Fernandes E. K., Keyser C. A., Hallsworth J. E., 2015 Stress tolerance and virulence of insect-pathogenic fungi are determined by environmental conditions during conidial formation. Curr. Genet. 61: 383–404. 10.1007/s00294-015-0477-y 25791499

[bib33] Rangel D. E., Fernandes E. K., Braga G. U., and Roberts D. W., 2011 Visible light during mycelial growth and conidiation of *Metarhizium robertsii* produces conidia with increased stress tolerance. FEMS Microbiol. Lett. 315: 81–86. 10.1111/j.1574-6968.2010.02168.x 21204917

[bib34] Ritchie M. E., Phipson B., Wu D., Hu Y., Law C. W., 2015 limma powers differential expression analyses for RNA-sequencing and microarray studies. Nucleic Acids Res. 43: e47 10.1093/nar/gkv007 25605792 PMC4402510

[bib35] Ruger-Herreros C., Rodríguez-Romero J., Fernández-Barranco R., Olmedo M., Fischer R., 2011 Regulation of Conidiation by Light in *Aspergillus nidulans*. Genetics 188: 809–822. 10.1534/genetics.111.130096 21624998 PMC3176103

[bib36] Saeed A. I., Sharov V., White J., Li J., Liang W., 2003 TM4: a free, open-source system for microarray data management and analysis. Biotechniques 34: 374–378. 10.2144/03342mt01 12613259

[bib37] Saini P., Eyler D. E., Green R., and Dever T. E., 2009 Hypusine-containing Protein eIF5A Promotes Translation Elongation. Nature 459: 118–121. 10.1038/nature08034 19424157 PMC3140696

[bib38] Sancar G., Sancar C., Brugger B., Ha N., Sachsenheimer T., 2011 A global circadian repressor controls antiphasic expression of metabolic genes in *Neurospora*. Mol. Cell 44: 687–697. 10.1016/j.molcel.2011.10.019 22152473

[bib39] Schumacher J., Simon A., Cohrs K. C., Viaud M., and Tudzynski P., 2014 The transcription factor BcLTF1 regulates virulence and light responses in the necrotrophic plant pathogen *Botrytis cinerea*. PLoS Genet. 10: e1004040 10.1371/journal.pgen.1004040 24415947 PMC3886904

[bib40] Shi Z., Fujii K., Kovary K.M., Genuth N.R., Röst H.L., 2017 Heterogeneous ribosomes preferentially translate distinct subpools of mRNAs genome-wide. *Mol Cell* 67 :71–83 e77. 10.1016/j.molcel.2017.05.021 PMC5548184 28625553

[bib41] Smith M. D., Gu Y., Querol-Audí J., Vogan J. M., Nitido A., 2013 Human-Like Eukaryotic Translation Initiation Factor 3 from *Neurospora crassa*. PLoS One 8: e78715 10.1371/journal.pone.0078715 24250809 PMC3826745

[bib42] Spriggs K. A., Bushell M., and Willis A. E., 2010 Translational regulation of gene expression during conditions of cell stress. Mol. Cell 40: 228–237. 10.1016/j.molcel.2010.09.028 20965418

[bib43] Trapnell C., Hendrickson D. G., Sauvageau M., Goff L., Rinn J. L., 2013 Differential analysis of gene regulation at transcript resolution with RNA-seq. Nat. Biotechnol. 31: 46–53. 10.1038/nbt.2450 23222703 PMC3869392

[bib44] Trapnell C., Roberts A., Goff L., Pertea G., Kim D., 2012 Differential gene and transcript expression analysis of RNA-seq experiments with TopHat and Cufflinks. Nat. Protoc. 7: 562–578. Erratum 9: 2513. 10.1038/nprot.2012.016 22383036 PMC3334321

[bib45] Trapnell C., Williams B. A., Pertea G., Mortazavi A., Kwan G., 2010 Transcript assembly and quantification by RNA-Seq reveals unannotated transcripts and isoform switching during cell differentiation. Nat. Biotechnol. 28: 511–515. 10.1038/nbt.1621 20436464 PMC3146043

[bib46] Wessel D., and Flugge U. I., 1984 A method for the quantitative recovery of protein in dilute solution in the presence of detergents and lipids. Anal. Biochem. 138: 141–143. 10.1016/0003-2697(84)90782-6 6731838

[bib47] Wu C., Yang F., Smith K. M., Peterson M., Dekhang R., 2014 Genome-Wide Characterization of Light-Regulated Genes in *Neurospora crassa*. G3 (Bethesda) 4: 1731–1745. 10.1534/g3.114.012617 25053707 PMC4169166

[bib48] Yamasaki S., and Anderson P., 2008 Reprogramming mRNA translation during stress. Curr. Opin. Cell Biol. 20: 222–226. 10.1016/j.ceb.2008.01.013 18356035 PMC2841789

[bib49] Yu Z., and Fischer R., 2019 Light sensing and responses in fungi. Nat. Rev. Microbiol. 17: 25–36. 10.1038/s41579-018-0109-x 30377305

[bib50] Zhou C., Arslan F., Wee S., Krishnan S., Ivanov A. R., 2005 PCI proteins eIF3e and eIF3m define distinct translation initiation factor 3 complexes. BMC Biol. 3: 14 10.1186/1741-7007-3-14 15904532 PMC1173091

